# High Resolution Genome-Wide Analysis of Chromosomal Alterations in Burkitt's Lymphoma

**DOI:** 10.1371/journal.pone.0007089

**Published:** 2009-09-17

**Authors:** Saloua Toujani, Philippe Dessen, Nathalie Ithzar, Gisèle Danglot, Catherine Richon, Yegor Vassetzky, Thomas Robert, Vladimir Lazar, Jacques Bosq, Lydie Da Costa, Christine Pérot, Vincent Ribrag, Catherine Patte, Jöelle Wiels, Alain Bernheim

**Affiliations:** 1 CNRS, FRE2939, Génomique Cellulaire des Cancers, Institut Gustave Roussy (IGR), Villejuif, France; 2 Université Paris-Sud, Orsay, France; 3 IGR, Functional Genomics Unit, Villejuif, France; 4 CNRS, UMR 8126, IGR, Villejuif, France; 5 IGR, IFR 54, Villejuif, France; 6 Cytogenetic Laboratory, Hôpital Saint Antoine, Paris, France; Health Canada, Canada

## Abstract

Additional chromosomal abnormalities are currently detected in Burkitt's lymphoma. They play major roles in the progression of BL and in prognosis. The genes involved remain elusive. A whole-genome oligonucleotide array CGH analysis correlated with karyotype and FISH was performed in a set of 27 Burkitt's lymphoma-derived cell lines and primary tumors. More than half of the 145 CNAs<2 Mb were mapped to Mendelian CNVs, including GSTT1, glutathione s-transferase and BIRC6, an anti-apoptotic protein, possibly predisposing to some cancers. Somatic cell line-specific CNVs localized to the *IG* locus were consistently observed with the 244 K aCGH platform. Among 136 CNAs >2 Mb, gains were found in 1q (12/27), 13q (7/27), 7q (6/27), 8q(4/27), 2p (3/27), 11q (2/27) and 15q (2/27). Losses were found in 3p (5/27), 4p (4/27), 4q (4/27), 9p (4/27), 13q (4/27), 6p (3/27), 17p (3/27), 6q (2/27),11pterp13 (2/27) and 14q12q21.3 (2/27). Twenty one minimal critical regions (MCR), (range 0.04–71.36 Mb), were delineated in tumors and cell lines. Three MCRs were localized to 1q. The proximal one was mapped to 1q21.1q25.2 with a 6.3 Mb amplicon (1q21.1q21.3) harboring *BCA2* and *PIAS3*. In the other 2 MCRs, 1q32.1 and 1q44, *MDM4* and *AKT3* appeared as possible drivers of these gains respectively. The 13q31.3q32.1 <89.58–96.81> MCR contained an amplicon and *ABCC4* might be the driver of this amplicon. The 40 Kb 2p16.1 <60.96–61> MCR was the smallest gained MCR and specifically encompassed the *REL* oncogene which is already implicated in B cell lymphomas. The most frequently deleted MCR was 3p14.1 <60.43–60.53> that removed the fifth exon of *FHIT*. Further investigations which combined gene expression and functional studies are essential to understand the lymphomagenesis mechanism and for the development of more effective, targeted therapeutic strategies.

## Introduction

First described by D. Burkitt in 1958, Burkitt's lymphoma (BL) is a monoclonal proliferation of malignant B lymphocytes. It is a mature aggressive lymphoma that accounts for 30 to 50% of lymphomas in children and for 1 to 2% in adults [1]. Its original description, a jaw tumor, led to the discovery of Epstein-Barr virus (EBV). Because of its high frequency in children living in the African equatorial regions (the Burkitt Belt), heavily infected by EBV and malaria [2] this viral linked tumor was qualified of endemic. It was identical, by morphology (cytology and pathology), immunophenotype and chromosome abnormalities to mature aggressive lymphomas, mainly EBV negative, that were observed in all the world. This type of BL, coined as sporadic, accounts for 30 to 50% of lymphomas in children and for 1 to 2% in adults [1]. The treatment of endemic and sporadic BL rely on high dose of chemotherapy with good results in children.

The hallmark of this disease is the t(8;14) translocation or one of the variant t(8;22) or t(2;8) translocations, associating the *MYC* gene (located at 8q24) to one of three immunoglobulin loci [3], [4]. These balanced chromosomal translocations result in constitutively deregulated *MYC* expression by position effect, whatever the break point location and the transcription orientation [5], Hypothesized as a transforming factor located on 8q24, MYC was the first oncogene isolated in a human malignancy, responsible for maintaining the balance of cellular proliferation, differentiation, adhesion and apoptosis.

By virtue of the translocation, MYC is in the position of the variable part of Ig genes [5] that is subjected to hypermutation by the AID activation [6].

Additional chromosomal abnormalities, detected in 70% of pediatric BL, seem to be important factors influencing clinical outcome [7], [8]. Gain of 7q and del(13q) seems strong genetic indicators of a poor prognosis [9]. Although many of these chromosomal anomalies undoubtedly play major roles in the pathogenesis of BL, the genes involved have remained elusive.

High resolution CGH array (aCGH) [10], [11] is a powerful method to identify pathogenic DNA copy-number changes (gain, amplification and deletion) on a genome-wide scale, and to map these changes to genomic sequence. It does not detect balanced structural rearrangements such as translocations. It is based on isolation of genomic DNA isolated from test and reference cell populations, differential labeling with fluorescent dyes and cohybridization with a high-resolution DNA microarrays. Oligonucleotide CGH arrays enable genome-wide detection of DNA copy-number changes down to 15∼20 Kb.

Recurrent chromosomal rearrangements in various tumors define Minimal Critical Regions (MCR) that are often small enough to pinpoint the few candidate genes in oncogenesis that lie in this chromosomal region. Many of these MCR contain known oncogenes and tumor suppressor genes and also help identify new genes that are critical in lymphomagenesis.

In this study, whole-genome 44K and 244K oligonucleotide arrays were used for fine mapping of recurrent copy number alterations (CNA) present in a set of 27 human Burkitt's lymphomas. These results allow a global view of genomic instability at a high level resolution (50 Kb on average) and the identification of new gene loci that are recurrently altered.

## Materials and Methods

### Primary Tumors and Cell Lines

The patients were studied according to various protocols approved by the French Ethics Committees. Between 1995 and 2005, 13 BL tumors were collected and frozen at disease onset. Tumor specimens were reviewed by an expert hematopathologist according to current WHO criteria for morphologic, immunophenotypic, and cytogenetic features.

Fifteen Burkitt's lymphoma-derived B cell lines were studied: BL2, BL31, BL41, BL70, BL84, BL104, BLLAL, Ly47, Ly91, Namalwa, Ramos, Oku, Salina, Seraphina and BLMer (derived from the relapse of tumor case 29124, thus counting one BL for both samples). They were cultured at 37°C in complete medium (GIBCO-BRL, Scotland) containing 2 mM L-glutamine, 1 mM pyruvate, 20 mM glucose, 20 µg/ml gentamicine and supplemented with 5% heat-inactivated fetal calf serum.

### Ethics Statement

No ethics statements are required for this work.

### Cytogenetic analysis

Cytogenetic analysis was performed on metaphase spreads obtained from tumor biopsy or bone marrow specimens and cell lines. In each case, 15 RHG-banded metaphases were analyzed when possible. Clonal chromosomal abnormalities were described according to the International System for Human Cytogenetic Nomenclature [12].

### Fluorescence in situ hybridization (FISH) studies

A set of commercial probes was used to diagnosis in some cases and to resolve discrepancies between aCGH results and karyotype data (Supplementary Table S1). To confirm DNA copy number changes obtained from aCGH, a FISH analysis was carried out using individual probes on cytogenetic preparations. Five BAC clones (Table S1) were selected from Pieter De Jong libraries (http://www.chori.org/bacpac/) according to their position on the UCSC human genome (http://www.genome.ucsc.edu/). Purified BAC DNA was labeled by random priming in the presence of Alexa 488-dUTP (green) and Alexa 594-dUTP (red) (Abbott). The quality of the probes was verified by hybridization to metaphase spreads of a negative control. The preparations were observed with an epifluorescence microscope and images captured with a Vysis imaging station. Between 20 and 40 metaphase spreads and 100 nuclei were analyzed for each sample.

### Oligonucleotide aCGH

Tumor genomic DNA was isolated according to Qiagen protocols with modifications [13]. Samples containing a t(8;V) were detected by cytogenetic analysis and/or FISH (dissociation of the MYC signal) in more than 60% of cells. High-molecular-weight genomic DNA was extracted from the cell lines with a DNeasy extraction kit (Qiagen).

Patient tumor samples and cell lines were analyzed using 44K and 244K microarrays (Agilent Technologies, Santa Clara, CA, USA) respectively. Only cell lines were processed as dye-swap pairs. In all experiments, sex-matched DNA from a pooled human female or male individual (Promega, Madison, WI) was used as the reference. Oligonucleotide aCGH processing was performed as detailed in the manufacturer's protocol (version 4.0; http://www.agilent.com). Data were extracted from scanned images using feature extraction software (version A.8.5.3, Agilent). Raw data text files from the latter were then imported for analysis into CGH Analytics 3.4.40. Aberrations were detected with the ADM2 algorithm and filtering options of a minimum of 5 probes and abs(log2Ratio) >0.3. Aberration segments were individually reviewed using build 35, hg17 of UCSC [14]. Anomalies that were localized to regions with high-copy repetitive or GC-rich DNA sequences including telomeric regions were excluded. We defined gains and losses for the oligonucleotide dataset as a linear ratio ≥1.2 or ≤0.8 respectively. High and low-level amplification events were defined as a linear ratio ≥4 or 2< ratio <4 respectively. The data are described in accordance with MIAME guidelines and have been deposited in ArrayExpress under E-TABM-703 accession number.

## Results

### Cytogenetic results

The karyotype of the primary tumor was complex in 4 cases (Table 1).

**Table 1:** Small and large scale chromosomal imbalances in Burkitt tumors.

Tumors	Age (years)/Sex	CNA	Coordinates (Mb)	Cytogenomic karyotype
28787	4/F	−2p23.1p16.3	<31.32–49.17>	46,XX,der(3)t(3;?)(q2;?),t(8;14)(q24.2;q32.3)[15]
		−3q26.3q27.1#	<183.94–185.30>	
		+7q21.1qter	<87.24–158.6>	
		−12q21.3#	<79.246–80.19>	
29123	8/M	+1q21.1q25.3	<143.72–182.28>	47,XY,dup(1)(q21q25),t(8;14)(q24.2;q32.3),
		−13q21.1q21.2	<52.77–57.69>	del(17)(p11),+ mar[15]
		+13q21.3q33.1	<69.49–103.22>	
		−13q33.1q34	<103.63–109.46>	
		−17pterq11.1	<0.04–22.33>	
29124	32/M	no statistically significant CNA		46,XY,t(8;14)(q24.2;q32.3)[150]
29125	68/F	−1p31.1p12	<71.21–119.36>	47∼49,XX,del(1)(p13p31),t(2;8)(p11.2;q24.2),
		+1q21.1q32.1	<148.08–205.17	der(3)t(3;?)(q27;?),−9,+ add(11)(p15),+ mars[15]
		−3q28qter	<190.07–194.87>	
		−4p16.1p14	<10.88–37.52>	
		−4q32.1qter	<161.36–191.12>	
		−9pterp13.3	<0.32–33.06>	
		−9q12q34.1	<66.22–129.48>	
		−11pterp13	<3.3–32.41>	
		+11q22.1qter	<101.45–133.95>	
		−13q14.1q14.3	<40.4–51.07>	
29127	9/M	no statistically significant CNA		ish t(8;14)(q24.2;q32.3)(wcp8+,wcp14+; wcp14+,wcp8+)
29139	82/M	−1p36.3p35.3	<3.64–29.63>	47,XY,der(3)del(3)(p?)t(3;?)(?;?),t(8;14)
		−3pterp11.1	<0.22–90.26>	(q24.2;q32.3.3),+mar[15]
		+3q11.2qter	<95.29–199.28>	
		−6q13q22.1	<75.21–114.87>	
		−9p21.3[x0.25] #	<21.73–22.31>	
		+10pterp14	<0.13–9.39>	
		−11q13.5qter	<75.18–133.72>	
		−13q12.1q14.1	<20.35–42.57>	
		−13q21.2q31.3	<59.28–93.42>	
		−13q32.3qter	<99.06–112.61>	
		−14q12q21.3	<26.52–43.84>	
		+18q11.1qter	<16.9–76.08>	
29140	12/M	no statistically significant CNA		46,XY,t(8;22)(q24.2;q11.2)[15]
29141	41/M	+1q21.1q25.3	<144.28–181.75>	46,XY,t(8;14)(q24.2;q32.3),der(13)t(1;13)(?;?)[15]
		−13q31.1q31.2	<84.36–86.95>	
		−13q33.1qter	<103.63–114.07>	
29142	61/F	no statistically significant CNA		46,XX,t(8;14)(q24.2;q32.3)[15]/idem,del(4)(q24q31)[4]
29143	62/M	no statistically significant CNA		46,XY,t(8;14)(q24.2;q32.3)[15]
29145	57/M	+1q21.1q25.3	<143.72–178.73>	46,XY,dup(1)(q21q25),der(14)t(8;14)(q24.2;q32.3)[15]
		+8q24.2qter	<128.81–146.25>	
		−11q24.1qter	<120.99–133.76>	
29146	55/M	no statistically significant CNA		46,XY,t(8;14)(q24.2;q32.3)[15]
29147	15/M	+2p16.1#	<60.96–61>	46,XY,t(2;8)(p11.2;q24.2),add(13)(q?)[15]
		+8p23.1#	<10.1–11>	
		+13q31.3q32.3	<89.58–99.61>	
		−13q32.3qter	<99.87–114.07>	

Results from chromosomal banding are in plain characters and # indicate small size CNAs.

Among the 15 cell lines, 13 exhibited a complex karyotype (Table 2); 11 harbored the same chromosomal abnormalities that were previously described [15], [16], while BLLAL, BLMER, OKU and Salina have not been reported elsewhere. The hallmark t(8;14) translocation was detected in 18 tumors or cell lines. The variant t(8;22) translocations were found in 1 tumor and 5 cell lines and the t(2;8) were found in 2 tumors and 1 cell line.

**Table 2:** Small and large scale chromosomal imbalances in Burkitt cell lines.

Cell Lines	CNA	Coordinates (Mb)	Cytogenomic karyotype
BL2	+1q21.1q31.3	<142.11–193.88>	46,XY,der(1)dup(1)(q**21.1q31**.3)dic(1;7)(**q31.3**;**q10**),
	−6q24.1qter	<142.01–170.82>	dic(1;6)(**q10;q24.1**),t(8;22)(q24.2;q11.2),
	+ 7q11.2qter	<61.47–158.57>	*del(10)(p12.1)*,der(13;13)(q10;q10)[15]
	−9p21.3[x0,05]#	<21.89–22.54>	
	−10pterp12.1	<0.11–38.66>	
	+13		
	−15q14#	<34.61–35.52>	
BL31	+1q21.1q31.1	<142.56–184.06>	46,XY,dup(1)(*q21.1q31.1*),t(8;14)(q24.2;q32.3),
	−10p14p11.2	<11.25–36.95>	*del(10)(p14p11.2)*,*der(13)del(13)(q13.1q31.2)*
	−13q13.1q31.2	<32.35–87.94>	*dup(13)(q31.2qter)* [15]
	+13q31.2qter	<88.01–112.92>	
BL41 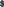	+1q12q21.1	<141.46–142.87>[x2.6]	84∼89,XXYY,*dup(1)(p36.1p35.1),dup(1)(q12q25.3),*
	+1q21.1	<142.87–143.28>[x3.8]	*del(2)(p16.3q36.3)*,del(3)*(q13.1q21.3)*x2,−4,
	+1q21.1q25.3	<143.28–180.03>[x2.6]	der(4)(4pter->q32.2::?->?::->4q35.1->4qter),
	+1q32.1#	<200.97–201.36>	del(6)*(6p12.3q12)*,+7x2,−8,
	+2p16.1p15	<60.82–62.21>[x3]	t(8;14)(q24.2;q31.3)x2,*del(9)(p24.1q22.1)*,
	+2p15p14	<62,22–65,11>[x1,8]	del(11)(p15.1q22.3),*del(16)(p11.2q23.2)*,
	−2p12qter	<79,22–230,59>[x0,75]	**del(17)(** ***p11.2*** **)x2,** *dup(17)(q21.3qter)*,
	−3q13.1q21.3	<106,02–131,82>	***del(18)(p11.31q22.3)*** **,**+20,
	−3q26.3	<176,02–178,83>	***der(22)t*** *(11;22)(q22.3;q13.3),mar(s)* [15]
	−4p16.2q13.3	<4,92–71,26>[x0,75]	
	−4q22.1q35.1	<90,69–187,4>	
	−5q33.1#	<150,42–151,29>	
	−5q33.2#	<154,144–155,125>	
	−6pterq12	<0,1–67,66>[x0,75]	
	+7		
	−8		
	−9p24.1p21.1	<7,5–32,13>[x0,75]	
	−9p13.2q33.1	<37,92–119,58>[x0,75]	
	−11		
	+12p13.3#	<5,88–7,27>	
	−15q14#	<33,47–33,5>	
	−16		
	−17pterp11.2	<0,02–19,68>	
	+17p11.2#	<19,69–21,44>	
	+17q21.3qter	<41,06–78,65>	
	−18		
	+20		
	−22q13.3qter	<44,82–49,5>	
BL70	+1q21.2q25.2	<146,81–176,59>	47,XY,dup(1)(q21.2q25.2),del(2)*(q13q24.1)*,
	−2q13q24.1	<110,18–156,88>	+7,t(8;14)(q24.2;q32.3),t(12;22)(q12;**q13**)[15]
	+7		
BL84	+1		50,XY,+Y,+1,der(3)del(3)(p22.1p12.3)*del(3)*
	−3p22.1p12.3	<41,97–76,58>	*(q26.2q28),*+5,t(8;22)(q24.2;q11.2),+der(8)t(8;22)
	−3q26.2#	<172,08–172,15>	(q24.2;q11.2)[15]
	−3q26.3q28	<184,06–191,84>	
	+5		
	+8pterq24.2	<0,06–129,15>	
	+22q11.2q13.3	<21,58–49,05>	
	+Y		
BL104	−4q13.2q21.1	<67,38–77,21>	47,XX,t(1;3)(q24;p25),**t(8;12)(p23;q24),**
	−4q28.3q31.2*	<136,56–151,65>	t(8;22)(q24.2;q11.2),+**inv(12)(?p13q24.2)** [8]/
	−4q34.1#	<174,14–174,94>	47,XX,t(1;3),***der(4)del(4)(q13.2q21.1)t(4;?)(q21.1;?),***
	−4q34.3q35.1	<182,3–186,69>	***der(4)del(4)(q28.3q35.1)t(4;?;12)(q35.1;?;?),***
	−5p13.2#	<36–37,44>	***t(8;22),der(9)del(9)(q21.3q31.1)t(4;9;12;?)(?;?q31;?;?)***
	−9q21.3q31.1	<79,24–100,39>	**,** ***der(12)t(5;12)(q13.2;?),+der(12)(4;5;12)(?;?;?)*** [7]
	+12		
BL LAL	no statistically significant CNA		46,XY,t(8;14)(q24.2;q32.3),t(12;22)(q12;q**11.2**)[15]
BLMer	−3p14.2#	<60,28–60,64>	47,XY,t(5;22)(q12;q**13**),t(8;14)(q24.2;q32.3),
	−5q11.2q12.13#	<58,88–59,12>	+13,der(18)t(13;18)(q**12.2;**q**21.2**),
	+13q12.2qter [x2]	<27,74–114,12>	*der(19)del(19)(q13.2qter)dup(19)(q13.1q13.2)* [15]
	−18q21.2qter	<51,67–76,11>	
	+19q13.1q13.2	<40,69–46,22>	
	−19q13.2qter	<46,54–63,78>	
Ly47	+1q43q44	<236,62–245,43>	47,XY,+Y,*dup(1)(q43q44)*,*der(6)t(3;6)(6pter->6q24.1:*
	−3p14.2#	<60,36–60,54>	*:3q26.3->3qter)*,***der(8)(8pter->8q24.2::22q11.2>22q12.3***
	+3q26.3qter	<176,05–199,38>	***:8q21.2.->8q24.2::22q11.2->22qter),***
	−6q24.1qter	<142,16–170,83>	***der(11)(11pter->11q2?3::16q21->16qter),***
	+8q21.2q24.2	<86,91–129,16>	del(14)*(q31.3qter)*, **der(18)(11q2?3::18p11->18qter),**
	−14q31.3qter	<88,09–106,34>	*der(22)t(8;22)(q24.2;q11.2)* [15]
	+16q21qter	<62,92–88,67>	
	+22q11.2q12.3	<21,58–31,49>	
	+Y		
LY91	no statistically significant CNA		46,XX,t(2;8)(p11.2;q24.2)[12]/46,XX,idem,
			*der(13)t(* ***1*** *;13* ***)(q32.1;*** *p12)* [3]
Namalwa	+1pterp36.1	<1,74–18,8>	45X,?-Y,*dup(1)(pterp36.1)*,dup(1)(*q11q31.1*),
	+1q12q31.1	<141,5–186,86>	der(3)t(3;4)(*3qter->3p11.1::4p11->4qter*),
	+1q44#	<240,24–241,35>	der(3)(**3pter**->**3q29::5q12.1->5q23**),-4,
	−3p25.1p11.1	<15,3–90,39>	der(5)**(5pter->5q12.1::5q23->qter),**
	+3q26.1qter	<163,87–199,32>	der(6)t(3;6)(3q26.3;6p22.2),+7, *dup(9)(q34.1qter)*,
	−4pterp11	<0,62–48,92>	***der(10)del(10)(q22.1q23.1)t(3;10)(q28;p24)*** **,**
	−5q11.2q12.1	<53,99–61,52>	***der(14)del(14)(q12q24)t(8;14)(q24.2;q32.3)*** **,**
	−6pterp22.2	<0,33–24,68>	***der(17)t(3;13;17)(3p?ter::17p12::hsr13q22->q32:***
	+7		***:17p11.2->17qter)*** *,dup(18q21.1q21.3)* [17]
	+9q34.1qter	<127,34–138,34>	
	−10q22.1q23.1	<71,66–83,88>	
	+13q22.3q31.2	<76,54–92,10>[x4]	
	+13q31.3q32.1	<92,11–95,82>[x5,7]	
	−14q12q24.3	<31,73–75,3>	
	+17p12p11.2	<15,47–21,1>	
	+18q21.1q21.3	<44,13–55,48>	
OKU	+2pterq21.3	<0,02–136,96>	46,XY,+del(2)(q21.3),-6,t(8;22)(q24.2;q11.2)[5]/
	−8pterp12	<0,16–29,88>	47,XY,+del(2)(***q*** *21.3*),
	+10q22.1qter	<71,61–135,31>	***der(8)(22qter->22q11.2::8q24.2>8p12::10q22.1>10qter)*** **,**
	+15q26.2qter	<92,15–100,21>	der(8)t(8;15)(8qter->8p2?3::15q26.2->15qter)[11]
Ramos**	−3p14.2#	<60,43–60,53>	45,X,?-Y,del(3)*(q12.3q21.3)*,inv(4)(p14q21),
	−3q12.3q21.3	<102,69–129,51>	*del(6)(q22.3q23.2)*,t(8;14)(q24.2;q32.3),
	+3q23qter	<143,27–199,32>	der(16)t(7;16)*(q11.2;p13)*, ***der(17)(17qter->17p11.2:***
	−5q31.2#	<138,16–138,27>	***:hsr(13)(q14q31)*** **,**
	−6q22.3q23.2	<121,26–134,4>	***der(18)(18pter->18q21.3::18q12.1->18q21.3:***
	+7q11.2qter	<72,27–158,61>	***:3q23->3qter)***,+r [11]/44,idem,-r[4]
	+11q23.1#	<110,62–110,81>	
	−13q12.1q14.1	<25,14–39,85>	
	+13q14.1	<40,25–45,19>	
	+13q21.3q31.1	<70,41–85,36>	
	−13q31.1q31.2	<85,4–87,85>	
	+13q31.2q33.1	<87,87–101,55>	
	−13q33.1qter	<101,56–109,37>	
	−17pterp11.2	<0,02–20,58>	
	+18q12.1q21.3	<27,21–53,62>	
	−18q21.3qter	<52,65–76,11>	
	−Xq21.3	<88,85–92,1>	
Salina	+1q21.2q31.3	<146,66–192,35>	46,XX,dup(1)*(q21.2q31.1)*,t(8;14)(q24.2;q32.3)[9]/
	−4p15.3p15.2	<18,2–23,74>	46,sl,***der(4)t(4;10)(q34.3;q23.3)*** [4]/
	−4q34.3q35.2	<179,18–191,31>	46,Sdl1,der(4)t(4;?)(4p15.2;?)[2]
	+10q23.3q24.3	<96,97–105,61>	
Séraphina	+1q21.1qter	<142,57–245,43>	48,XX,der(2)t(1;2)
	+2pterp24.3	<0,02–14,02>	*(1qter->1q21.1::2p25->p24.3::2p25->2qter)*,+4,
	+4		der(6)*t(6;?)(?::6p22.1->6qter)*,t(8;14)(q24.2;q32.3),
	−5q33.3#	<158,08–158,18>	+15,*dup(16)(q23.2qter)*,*del(18)(p11.2)* [*15*]
	−6p24.2p22.1	<11,2–28,88>	
	+8q24.2#	<128,71–128,95>	
	+15		
	−18p11.3p11.2	<0–15,11>	
	+16q23.2qter	<79,59–88,69>	
	−20q11.2#	<32,51–32,65>	

* BL104 has a complex rearrangement on 4q.

** Ramos has a very complex rearrangement on 13q.

# indicate small size CNAs.

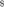
In BL41, the 1q12q25.2 gain was segmented due to the presence of two amplicons.

Results from chromosomal banding are in plain characters, those from FISH (see supplementary data table 1) are in bold and those from aCGH are in italic.

#### aCGH CNA correlate with conventional and molecular cytogenetic results

**Figure 1:**
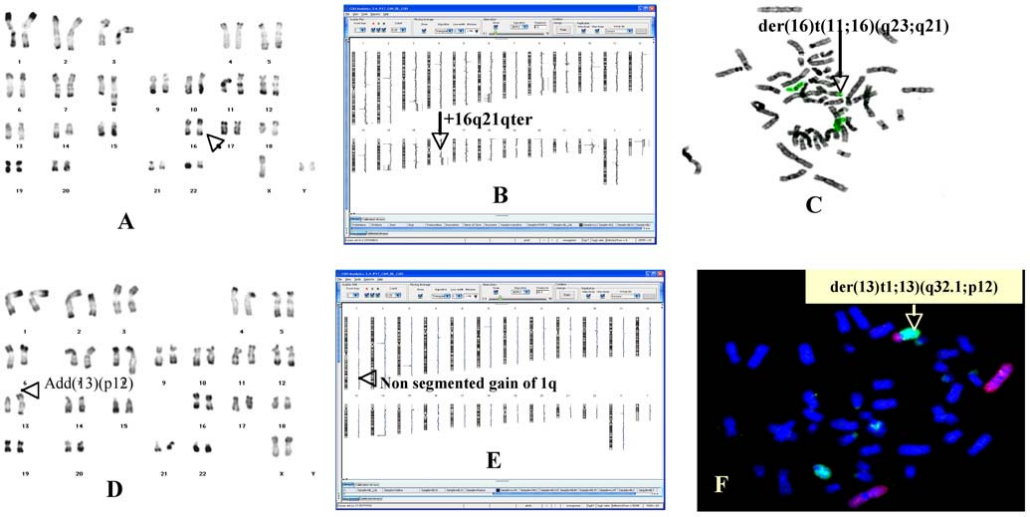
Correlation karyotype-high resolution aCGH. A RHG-banded karyotype of Ly47 showing 2 normal chromosome 16 (arrow). B Karyogramme of Ly47 cell line showing a gain of chromosome 16 (arrow). C FISH with whole painting of chromosome 16 confirmed aCGH result. The additional piece of chromosome 16 was translocated at the long arm of chromosome 11. D RHG-banded karyotype of Ly91 showing an add(13)(p12) which is presented in 20% of cells (arrow). E Karyogramme of Ly91 cell line showing a non segmented gain on 1q (arrow). F FISH with whole painting of chromosome 1(red) and 13(green) allowed to identify the origin of the material translocated at 13p. Hence, the add(13) became a der (13)t(1;13)(q32.1;p12).

A strong correlation between aCGH results and karyotype analysis was observed for CNA ≥5 Mb. Some larger CNA were detected in cell lines exclusively by aCGH. For example, in the Ly47 cell line, a gain of a 25.75 Mb region on 16q21qter <62.92–88.67> could not be detected by karyotype analysis (Fig. 1 A+B). This gain was confirmed by FISH (Fig. 1C). In the Ly91 cell line, a nonsegmented gain of 1q was observed on aCGH (Fig. 1E) that allowed FISH to identify a der(13)t(1;13)(q32.1;p12) present in only 20% of the cells (Fig. 1 D+F). Often aCGH refined the breakpoints detected by conventional cytogenetics. In addition to primary BL translocations, four other balanced translocations were only shown by karyotype analysis without any visible scar at 20 Kb resolution (Table 2).

### Analysis of small-scale copy number alteration: CNVs

Small sized CNA (n = 145) defined as ≤2 Mb long, ranging from 0.03 to 2 Mb in size, were detected in 140 occurrences originating from 244 K arrays, and 5 from 44 K. More than half of these small CNA were found to be Mendelian CNVs. This was tested by examining the sequence present at each CNA, with the University of California at Santa Cruz Genome Browser (http://genome.cse.ucsc.edu) and Toronto (http://projects.tcag.ca/variation) tools. Eighty one of the 145 small CNA were mapped to Mendelian CNV (mCNV) in the Database of Genomic Variants. Among these mCNV, there were very frequent CNVs overlapping the large group of olfactory receptor genes, the UGT2B17 and UGT2B28 genes. The genes encoding the HLA group were also detected within these common CNVs. In our set, the GSTT1 gene exhibited the most frequent CNVs. This locus was gained (5 copies) in 7 cases and homozygously lost in three cases.

### Immunoglobulin Somatic Copy Number Variation

**Figure 2:**
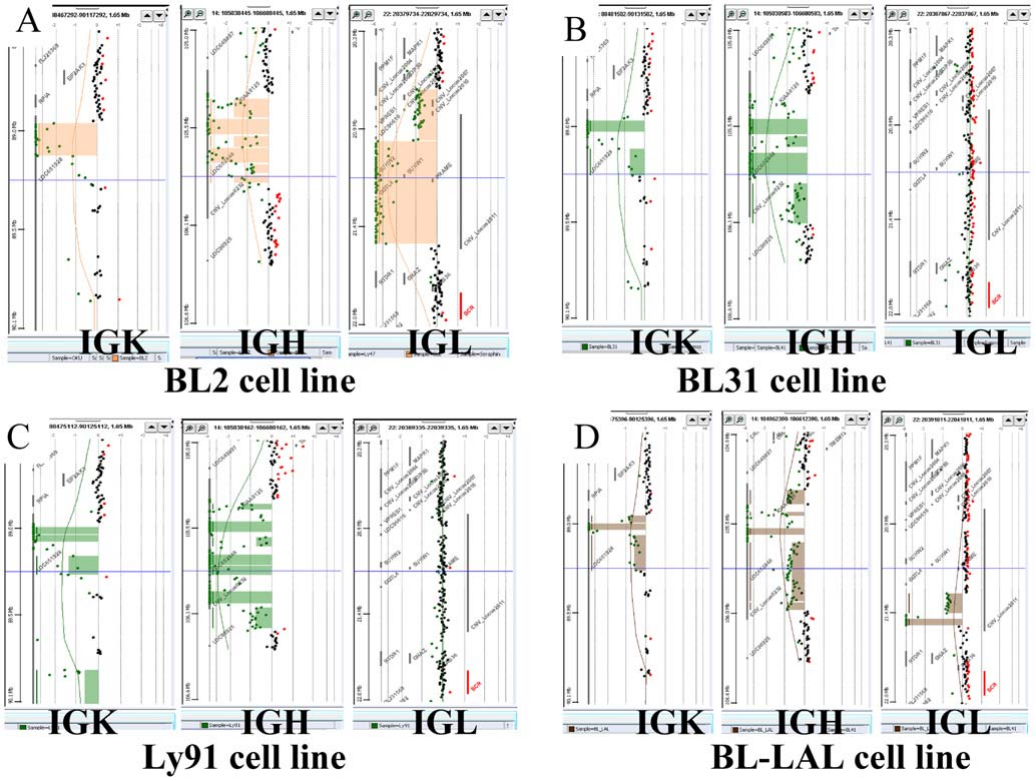
Immunoglobulin Somatic Copy Number Variation. The immunoglobulin loci at chromosomal subbands 2p11.2 (IGK), 14q32.3 (IGH) and 22q11.2 (IGL) showed acquired monoclonal alterations by the 244 K aCGH platform. The segmented rearrangements were identified by a stained rectangle and the individual oligonucleotides are identified by a bold dot. The Y axis is the chromosomal position, the X axis is the ratio (loss is to the left). A to D, S1: The IGH locus was rearranged in all cell lines (14/15) on the two chromosomes, with a specific pattern for each cell line. BL2 and BL31 have a t(8;14). A to D, S1: IGK gene rearrangements were detected in all cell lines and 8 have a clear biallelic rearrangement including LY91 the only t(2;8). A to D, S1: IGL loci somatic rearrangements were observed in only 10 cell lines (7 biallelics) including four cases of t(8;22) as in BLAL. Other cell lines are presented as supporting figures.

Thirty-eight of the small CNA were mapped to the immunoglobulin loci at chromosomal subbands 2p11.2 (IGK), 14q32.3 (IGH) and 22q11.2 (IGL) (Table S2, Figure 2, Figure S1, Figure S2, Figure S3, Figure S4, Figure S5, Figure S6, Figure S7, Figure S8, Figure S9, Figure S10, Figure S11). These acquired monoclonal alterations were ascertained only by the 244 K aCGH platform. The IGH locus was rearranged in all cell lines (14/15 on the two chromosomes), with a specific pattern for each cell line and it expressed surface IgM. In addition, IGK gene rearrangements were also detected in all cell lines (8/15 biallelic). IGK was homozygously rearranged in the only t(2;8). IGL locus somatic rearrangements were observed in 10 cell lines (7 biallelic). The other 5 cell lines, whose IGL locus was in germline configuration, only exhibited surface IgK expression (Table S2). In the t(8;22) the IGL gene was rearranged on the two alleles in 4 cases. The only hemizygote was observed in the OKU cell line (Figure S8) which expressed IGK. These results, consistent with the germinal center nature of BL cells, and the processes of maturation of immunoglobulin, show a very good concordance between immunophenotype and aCGH results.

### Other Small Somatic Copy Number Alterations

Among the 26 remaining small-scale CNA (Tables 1 and 2), 16 imbalances were localized to either intergenic regions or predicted gene sequences of unknown function. The significance of these CNA remains to be determined. Ten imbalances were mapped to regions containing characterized genes, many of which play roles in tumorigenesis and B-cell differentiation and proliferation. These genes allowed either the narrowing of the minimal critical regions or remained as a single CNA (see below).

### Analysis of large-scale copy number alteration

The total number of CNA >2 Mb was 136 in cell lines and tumors (Table 1). There were 17 whole chromosome gains or losses and 119 structural CNA (sCNA). The distribution of these sCNA was bimodal, with primary tumors or cell lines tending to have either a large number or no large CNA. From the frequency analysis, we observed the general trends of 1q (12/27), 13q (7/27), 7q (6/27), 8q (4/27), 2p (3/27), 11q (2/27) and 15q (2/27) gains whereas losses were more frequent at the following chromosomal regions: 3p (5/27), 4p (4/27), 4q (4/27), 9p (4/27), 13q(4/27), 6p (3/27), 17p (3/27), 6q (2/27), 11pterp13 (2/27) and 14q12q21.3 (2/27).

### Minimal critical region altered in BL

Some of these CNA were recurrent across different samples and allowed us to define 21 MCRs of gain/amplification or loss/deletion (Table 3). A nearly equal number of lost and gained MCR were defined and their median size was 4.96 Mb (range 0.04–71.36 Mb).

**Table 3:** Minimal critical regions issued from tumor and cell lines.

Cytogenetic band	position (Mb)	Size (Mb)	Gain/Loss	Cell lines	Tumors	Cancer-related genes in the region	miRNA
1q21.1q25.2	142,87–177	34,13	G	8	3	>300 genes, ***BCL9 PIAS3 BCA2 LHX4***	
1q32.1	200,97–201,36	0,39	G	3	0	*MDM4 PIK3C2B LRRN5*	
						*PLEKHA6 PPP1R15B*	
1q44	240,24–241,35	1,11	G	4	0	*ADSS HNRPU * ***AKT3*** * ZNF238*	
						*C1orf121 FAM36A C1orf100*	
						*C1orf101 LOC440742*	
2p16.1	60,96–61	0,04	AMP	2	1	***REL***	
3p14.2	60,43–60,53	0,1	L	4	1	***FHIT***	
4p15.3p15.2	18,2–23,74	5,54	L	3	1	*SLIT2 PPARGC1A GBA3 KCNIP4*	
						*MGC29898 GPR125*	
4q34.1	174,14–174,94	0,8	L	2	1	***HMGB2*** * SAP30 HAND2 MORF4*	
						*SCRG1 GALNT7 GALNT17*	
4q34.3q35.1	182,3–186,69	4,39	L	3	1	*SLC25A4 CASP3 DCTD ACSL1*	
						*ING2 IRF2 CARF LRP2BP STOX2*	
						*FLJ12716 MLF1IP WWC2 SNX25*	
						*ENPP6 FLJ30277 RWDD4A*	
						*FLJ33167 ANKRD37 HELT*	
6p24p22.4	11,2–26,4	15,2	L	3	0	>60 genes *BMP6 FOXC1 IRF4*	
						*MAK SSR1 TFAP2A RIPK1*	
						*PRPF4B EEF1E1 NRN1 EXOC2*	
						*WRNIP1 RIOK1 HUS1B CAGE1*	
6q24.1qter	142,16–170,83	28,67	L	2	0	135 genes, ***PDCD2***	
7q21.1qter	87,24–158,6	71,36	G	5	1	444 genes	
8q24.2	128,71–128,95	0,24	G	3	1	***MYC***	*mir-1204*
9p21.3	21,89–22,31	0,42	L	2	2	***CDKN2A/CDKN2B***	
11pterp13	pter-32,41	32,41	L	1	1	235 genes, ***FANCF,PLEKHA7***	
11q23.1	110,62–110,81	0,19	G	1	1	***POU2AF1*** * C11orf53 FLJ45803*	
13q31.3q32.1	89,58–96,81	7,23	AMP	5	2	***GPC5*** * DCT DNAJC3 CLDN10*	*mir-622 mir-17*
						*GPC6 * ***ABCC4 SOX21*** * DZIP1 TGDS*	*mir-18a mir-19a*
						*UGCGL2 GPR180 HS6ST3*	*mir-20a mir-19b-1*
							*mir-92-1*
13q32.2	97,79–97,85	0,06	G	4	2	***FARP1***	
13q33.1q34	103,64–112,61	9,3	L	0	3	***ING1 COL4A2 COL4A1 RAB20 LIG4***	
						***TNFSF13B*** * EFNB2 * ***ERCC5*** * FGF14*	
						*PCCA SLC10A2 IRS2 ARHGEF7*	
						*TNFSF13B ANKRD10*	
14q12q21.3	26,52–43,84	17,32	L	1	1	44 genes, ***FANCM***	*mir-624*
15q26.2qter	92,15–100,21	8,06	G	2	0	25 genes *MCTP2*, ***IGF1R***, *PCSK6*	
17pterp11.2	0–19,68	15,36	L	2	1	>200 genes ***TP53***	*mir-22 mir-132*
							*mir-212 mir-195*
							*mir-497 mir-324*

**Figure 3:**
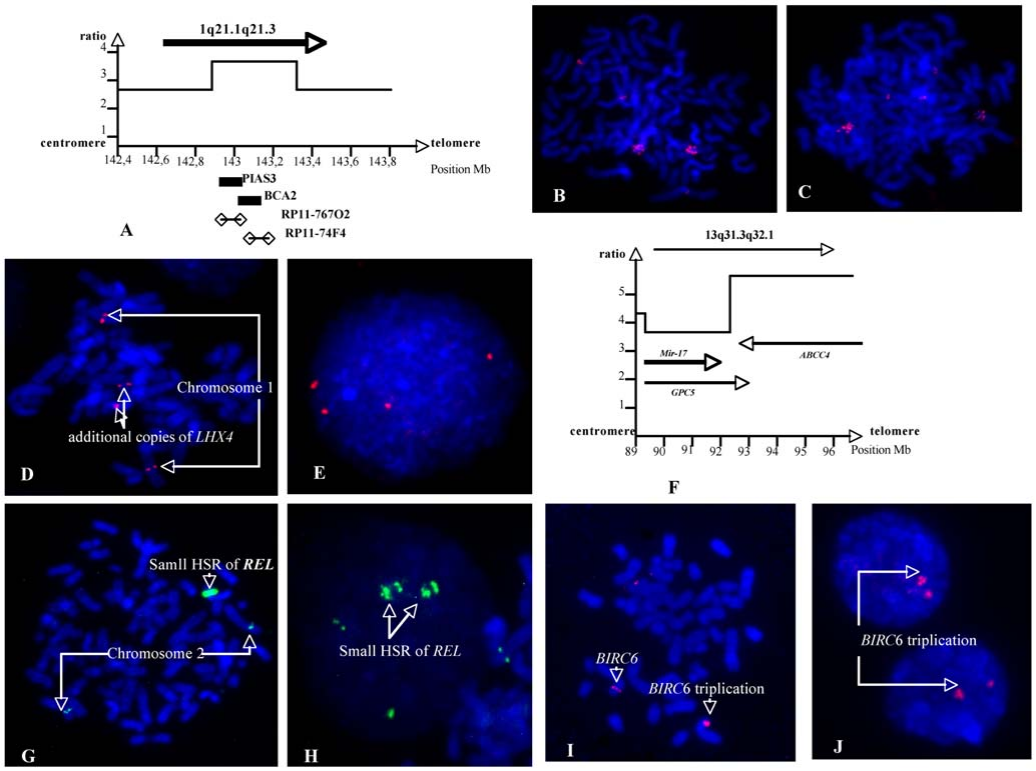
Various gained or amplified small regions description. A. Copy number profile of 1q21.1 amplicon in BL41:the 7 copies maximal amplitude region was the <142,87–143,28 Mb> interval that included *BCA2* and *PIAS3* genes. B and C. The amplifications of *BCA2* and *PIAS3* were confirmed by FISH with BAC clones RP11-767O2 and RP11-74F4 respectively which showed a small HSR. D and E. In Namalwa a low level amplification (4 to 5 copies) of 1q25.2 <176,6–177> was confirmed by FISH with BAC clone RP11-175C8 which contains the *LHX4* gene only. 3F. In Namalwa the 13q31.33q2.1 <89,58–95,82> region was amplified. The proximal part that harboured the polycistron mir-17 and a part of GPC5 gene was present in 8 copies. The distal region, with maximal amplitude (12 copies), contained 12 genes including GPC5, GPC6 and ABCC4 (see text). G and H. In BL 41 the *REL* gene was found amplified and FISH with BAC clone RP11-373L24 showed a small HSR. I and J Molecular cytogenetic on BLMer cell line showing a duplication of *BIRC6*. RP11-121M15 BAC clone was choosen on the region of *BIRC6*. The latter gene was duplicated as denoted by an arrow.

The most frequent gained region was 1q followed by 13q. Three different MCRs were mapped to 1q. The proximal one was mapped to 1q21.1q25.2. The BL 41 cell line exhibited a 6.3 Mb amplicon localized at 1q21.1q21.3 <142.87–149.17 Mb> (Fig. 3A+B+C). The region exhibiting the highest copy number (as many as 7 copies), contained 14 genes and among them, *BCA2* and *PIAS3* seemed to be possible oncogenes. Two other MCRs were isolated on 1q (Table 3). Three different MCRs were mapped to 13q (Table 3). The most frequent one mapped to 13q31.3q32.1, contained the Namalwa cell line amplicon (Fig. 3F). A 240 Kb MCR at 8q24.2 encompassed MYC. A loss of 13q33.1q34 was only found in tumors. In our set, the most frequent deleted region was 3p14.2 followed by 9p21.3 and a deletion of 17p (Table 3).

## Discussion

In this work, we report a fine mapping of chromosomal imbalances in a set of 27 BL primary tumors and cell lines. Whole-genome 44 K and 244 K oligonucleotide arrays were used to analyze recurrent copy number alterations present in tumors and cell lines respectively.

Comparison of the 15 dye-swap pairs from cell lines revealed identical aberrations. In most cases, the boundaries of the chromosomal aberrations were absolutely identical. The dye-swap helped to reduce the background level. Whole-genome oligonucleotide microarrays aCGH analysis allowed us to delineate the chromosomal imbalances at 15∼20 Kb resolution in the 15 cell lines (244 K) and at 70 Kb average resolution in the 13 primary tumors (44 K).

Each sample was also investigated using conventional cytogenetics. Close agreement between karyotype data and whole-genome aCGH CNAs was observed. When discrepancies occurred, they were mostly explained, *i*) by a lack of detection due to karyotype resolution; *ii*) by aCGH locally blurred heterogeneous cell clones, or normal cell contamination in the sample; *iii*) by the presence of balanced chromosomal rearrangements. In addition to BL hallmark anomalies, 4 other apparently balanced translocations were detected exclusively by conventional cytogenetics, as expected (Table 2). The karyotypes of the 11 previously published cell lines were essentially identical to those reported elsewhere [15], [16].

Four cell lines (BLLAL, OKU, SALINA, BLMer) have been karyotyped for the first time in the present paper. The BLMer cell line was established from a recurrent tumor (case 29124). Karyotype analysis of BLMer showed the same anomalies as observed in the parental tumor (*data not shown*). Although cell lines cannot fully recapitulate all the biological aspects of tumors [11], the chromosomal alterations observed in cell lines are representative of their parent histology [17].

In the primary tumor subgroup with CNA ≥1 (n = 6), the mean number of CNA per sample was 6 versus 9 for cell lines (n = 14), showing the same order of magnitude.

### Partial duplication of the PVT1 mi RNA locus: a role in BL?

**Figure 4:**
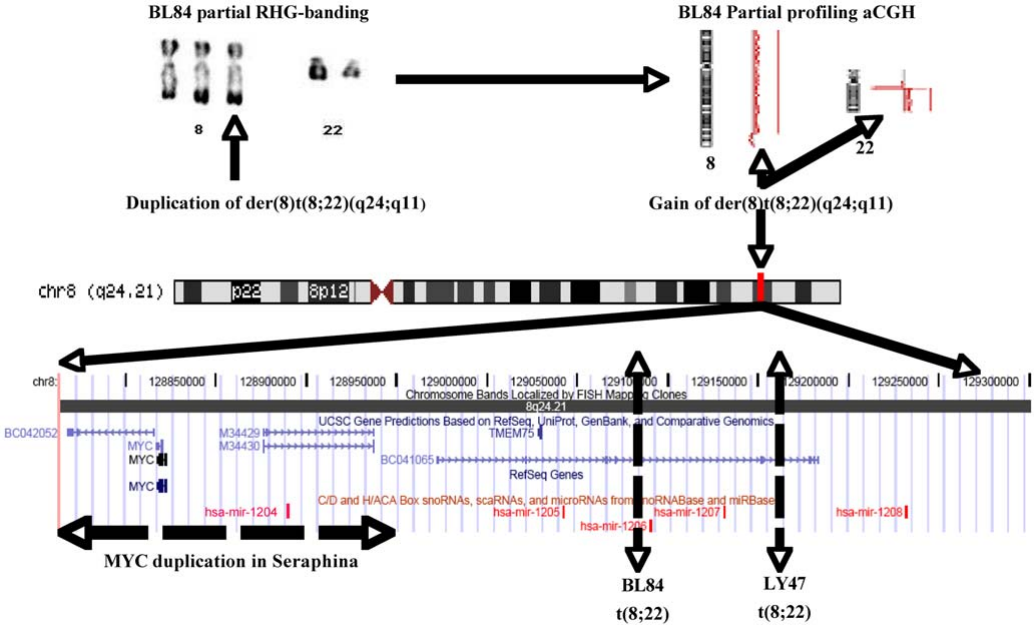
Virtual cloning of 8q24 region. *MYC* region breakpoints. In Seraphina, BL 84 and Ly 47 cell lines, *MYC* locus was gained. MYC region breakpoints vary with primary anomalies. In BL 84 and Ly 47 cell lines with t(8;22), MYC breakpoints were mapped to *PVT1*(Huppi et al., 2008); mir-1205 and mir1204 (not present on UCSC map, located on chr8:128877390-128877456) defined the MCR of this gain. In Seraphina, a 240 Kb duplication was observed around MYCand located by FISH in the immediate vicinity this gene on the der(14)t(8;14) (data not shown). This duplication contained hsa mir 1204.

In the BL 84 and Ly 47 cell lines, the der(8)t(8;22) was totally and partially duplicated respectively. This allowed the high-resolution mapping of the translocation. The MYC 3′ breakpoints were mapped to the *PVT1* locus that harbors *mir1204, mir1205, mir1206* and *mir1207* [18]. Among these genes, only *mir*1204 and *mir1205* were duplicated in the two cell lines (Fig. 4). The localization of these micro-RNAs in the 8q24 region raises questions. Are these micro-RNA implicated in lymphomagenesis. Are BL variant translocations a distinct group?

### CNAs and CNVs in BL

A total of 145 CNA ≤2 Mb were detected. Among them, there were 81 CNA mapped to Mendelian CNV. *GSTT1* was the most polymorphic gene in our set. *GSTT1* is a member of a multigenic family of detoxifying enzymes [19]. Four classes of isoenzymes have been linked to human malignancy: GSTM, GSTP, GSTA and GSTT. Several studies have shown that individuals who harbor *GSTM1^−/−^* or *GSTT1^−/−^* as well as *GSTP1* variants are at an increased risk for a series of tumors [19], [20]. Further studies are required to clarify its role in Burkitt's lymphoma.

CNV are not totally neutral variants. Many recent studies have directly and indirectly implicated some Mendelian CNV in genomic disorders [21], [22]. A 560 Kb low-level amplification of the 2p23 region was found in the BL Mer cell line and its parental tumor, and confirmed by FISH (Figure 3 I, J). This region harbors only *BIRC6* gene which encodes an inhibitor of apoptosis and a chimeric E2/E3 ubiquitin ligase [23]. However, the 3 copies on chromosome 2 and the single copy on the other chromosome have the same boundaries as a reported CNV. Interestingly, germline amplification of *BIRC6* was found in patients with a family history of pancreatic cancer [24].

Somatic CNVs mapped to the immunoglobulin locus [25], [26] were the second type found in our study. BLs have clonally rearranged *IGH, IGK* and *IGL* chain genes, hence these rearrangements can be detected by the 244 K aCGH platform that showed a high sensitivity between the oligonucleotide arrays [27]. The rearranged pattern is highly specific of each individual malignant cell line confirming that they are monoclonal proliferations. Several oligonucleotides have an intermediate value between one and two copy losses (Figure 2, Figure S1, Figure S2, Figure S3, Figure S4, Figure S5, Figure S6, Figure S9). This could be related to further rearrangements of IG genes in subclones. According to the genetic model of lymphomagenesis, the frozen differentiation stage might reflect the cell type in which the primary translocation t(8;14) took place leading to maturation arrest. An alternative hypothesis is that mature aggressive B-NHL originate from cells with stem-cell features or that stemness was acquired during lymphomagenesis by epigenetic remodeling [28].

### CNAs distribution

In our study, the distribution of the CNA >2 Mb was bimodal, with 6/12 primary tumors and 13/15 cell lines exhibiting additional abnormalities to the IG/MYC translocation, in accordance with the low number of chromosomal changes previously reported [29], [30]. A smaller group with partial uniparental disomy resulting in loss of heterozygosity without chromosomal imbalances besides classic chromosomal instability, has been reported in BL [29].

Gains were more frequent at the following regions 1q (12/27), 13q (7/27), 7q (6/27), 8q (4/27), 2p (3/27), 11q (2/27) and 15q (2/27). Ten MCRs were observed.

In BL, the most frequent additional anomaly is the duplication of 1q [8], [31].

### MCRs on chromosome 1

Three MCRs were observed on 1q. The major one was localized at 1q21.1q25.2 <142.87–177.00>. In a recent study using tiling-resolution aCGH, this MCR was larger and mapped to 1q12q25.2 [31]. In the BL 41 cell line, the 1q21.1q21.3 <142.87–149.17> region harbored a 6 Mb amplicon (Fig. 3A, B, C). In a recent study, a 1.35 Mb minimal deleted region <143.65–145.00>, was associated with developmental defects [32]. This region overlapped the BL41 amplicon, but the copy number peaks (<142.87–143.28>) seemed to be different. The genomic structure of 1q21 is extremely complex, with at least 4 large segmental-duplication blocks ranging in size from 270 Kb to 2.2 Mb. Consequently, these duplicons favored the non-allelic homologous recombination which might explain both the congenital anomalies (Mefford et al., 2008) and malignancy, particularly lymphoma. Although 1q rearrangements have no impact on the prognosis of BL [9], amplification does nevertheless contribute to tumor development [33]. *BCA2* and *PIAS3* that mapped at the maximal amplitude of the BL 41 amplicon are thus candidate driver genes in this amplicon. *BCA2* has E3 ubiquitin ligase activity and was found to be overexpressed in invasive breast cancer [34]. *PIAS3* codes for the Protein Inhibitor of Activated *STAT3*. *PIAS3* signaling has been shown to prevent apoptosis and enhance cellular proliferation through the regulation of genes such as *MYC* [35]. Overactivation of *STAT3* has been identified in many cancers [36].

The second 1q MCR was 1q32.1 <200.97–201.36>. It encompasses *MDM4.* This gene has not been implicated in BL, but *MDM4* inhibits *P53*, and in vivo development of B-cell lymphomas in Eμ-myc MDM4^+/−^ mice is delayed compared to that occurring in Eμ-myc MDM4^+/+^ mice [37].

The third 1q MCR was 1q44 <240.24–241.35>. It harbors *AKT3* which is one of the 3 isoforms of *AKT*. The latter is a Ser/Thr kinase in the PTEN/PI3K/AKT pathway and activation of *AKT* is often observed in human cancers [38]. In primary hepatocellular carcinoma, *AKT3* was up-regulated as a result of a gained 1q44 region [39]. In the Namalwa cell line, a 0.4 Mb low-level amplification (5 copies) at 1q25.2 <176.6–177> containing 5 genes was detected. Among them, *LHX4*, a LIM homeobox 4 was found to be amplified by FISH (Fig. 3D+E). *LHX4* is the partner of *IGH* in t(1;14)(q25;q32) detected in pre-B acute lymphoblastic leukemia [40].

### MCRs on other chromosomes

The second most gained arm was 13q. The major MCR was 13q31.3q32.1 <89.58–96.81> containing a 6.24 Mb amplicon <89.58–95.82> (Fig. 3F) as frequently seen in lymphomas but also across a broad range of tumor types [41]. The proximal segment of this amplicon contained microRNA-17 polycistron [42], [43] which was stably upregulated in the presence of constitutive *MYC* expression [44]. The highest copy number segment harbored 12 genes, including GPC5, GPC6 and ABCC4. Glypican genes (GPC5 and GPC6), belong to a family of heparan sulfate proteoglycans that are constantly expressed and up-regulated in rhabdomyosarcoma with an amplified 13q31q32 region [45]. However, in case 29147 and in Namalwa cell line, the DNA copy number of GPC5 argues against this gene as the driver of the amplification. *ABBC4* (MRP4), which encodes the multi-drug resistance protein, could also play this role. It was present in 12 copies in the Namalwa cell line. It was found to be amplified in several drug-resistant cell lines derived from various malignancies [46]. *ABBC4* transcripts were found upregulated in a set of BL with gain of 13q31q32 region [30].

On chromosome 8, *MYC* was the only gene contained in a 240 Kb gained MCR at 8q24.2, originating either from t(8;14) and t(8;22) rearrangements.

A 2p16 MCR contained a 40 Kb amplicon in BL 41. It harbored *REL* (Fig. 3G, H), a member of the NF-κB family of transcription factors, which was found to be amplified in primary mediastinal B-cell lymphoma [47].

The 11q23.1 MCR mapped to <110.62–110.81> contained *POU2AF1*, a B-cell specific transcriptional coactivator which is amplified in multiple myeloma [48].

A 1.8 Mb amplicon was found at 17p11.2, in the BL 41 cell line, with a 381 Kb peak of 10 copies <20.72–21.10> containing four genes of mostly unknown function. The proximal and distal boundaries of this amplicon with 7 copies, contained two kinases genes, *AKAP10* and *MAP2K3*. The genomic structure of 17p11 is complex, with several segmental-duplication blocks including one totaling 183 Kb.

### Gene loss regions in BL

**Figure 5:**
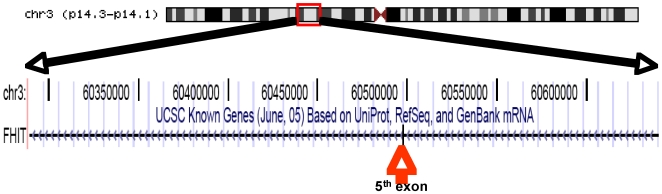
A small intragenic loss. Based on the UCSC data base (June, 2004), the 402 Kb MCR on 3p remove the fifth exon of FHIT gene.

The most frequent deleted arm was 3p in four cell lines and one tumor, a rare finding in BL. A 100 Kb MCR localized at 3p14.2 <60.43–60.53> contained the 5^th^ exon of *FHIT* (Fragile Histidine Triad) (Fig. 5), a finding in agreement with earlier studies [49].

On 9p, the 420 Kb MCR harbored *CDKN2A/CDKN2B*, a gene rarely reported to be impaired in BL [50].

On 13q33.1q34 a 10 Mb MCR, detected only in three tumors, harbored more than thirty genes. Among these *ING1, COL4A2, COL4A1, RAB20, LIG4, TNFSF13B*, all in a small region, could constitute the target of this loss. In case 29125 a single10 Mb deletion at 13q14.1q14.3 removed the critical mir-16-1 and mir-15a involved in CLL [42]. This rearrangement is not observed in the 15 cell lines.

On 17p, a large 19.86 Mb MCR had removed *P53* as well as several other genes. Mutations in the *P53* gene have been found in at least 33% of BL biopsy specimens [51] and in as much as 83% of BL cell lines [52], [53].

Some single deletions were observed. In case 28787, a deletion at 2p23.1p16.3 particularly removed *MSH2* and *MSH6* genes that do not seem to be frequently altered in lymphoma [54]. *EBF1* (5q33.3 <158.08–158.18>), the early B cell factor was lost in Seraphina. In a recent work on ALL, mono-allelic deletions of EBF1 were emphasized [55].

High resolution aCGH is a powerful method that allowed a fine mapping of additional unbalanced chromosomal abnormalities in BL, but karyotype still remain an essential tool to rapidly identify balanced chromosomal translocations. A subgroup of BL without CNAs, warrants further investigation (SNP array, whole genome sequence) in order to find the necessary additional oncogenic events to the MYC rearrangement [4]. The identification of the target genes of the large MCR will need correlations with other genomics data sets (gene expression, nucleotide sequence, epigenetic…) in order to make the low throughput functional gene studies.

With regard to additional chromosomal abnormalities studied by cytogenomics, BL appears to exhibit non-random genetic heterogeneity as revealed by this study. The MCRs remain to be fully functionally characterized in order to design targeted and personalized therapies in poor prognosis disease [9].

#### Data Availability

The microarray data analyzed in this paper have been submitted to the Array Express data repository at the European Bioinformatics Institute (http://www.ebi.ac.uk/arrayexpress/) under the following accession numbers: E-TABM-703.

## Supporting Information

Table S1 Commercial and BAC probes used to validate aCGH results
(0.07 MB DOC)

Table S2 Clonal Immunoglobulin phenotype linked to chromosomal abnormalities and acquired CNV
(0.08 MB DOC)

Figure S1 BL41 cell line.
(0.11 MB JPG)

Figure S2 BL70 cell line
(0.12 MB JPG)

Figure S3 BL84 cell line
(0.12 MB JPG)

Figure S4 BL104 cell line
(0.12 MB JPG)

Figure S5 BLMer cell line
(0.12 MB JPG)

Figure S6 Ly47 cell line
(0.11 MB JPG)

Figure S7 Namalwa cell line
(0.11 MB JPG)

Figure S8 OKU cell line
(0.12 MB JPG)

Figure S9 Ramos cell line
(0.12 MB JPG)

Figure S10 Salina cell line
(0.12 MB JPG)

Figure S11 Seraphina cell line
(0.12 MB JPG)
