# Supplementary material for: High Resolution Genome-Wide Analysis of Chromosomal Alterations in Burkitt's Lymphoma
Source: PLoS One. 2009 Sep 17;4(9):e7089. doi: 10.1371/journal.pone.0007089 (PMC2739276; doi:10.1371/journal.pone.0007089)
Supplement: Table S1 — Commercial and BAC probes used to validate aCGH results (0.07 MB DOC) [file pone.0007089.s001.doc]

| **Probe** | **chromosomal location** | **References** |
| --- | --- | --- |
| wcp(1) | chromosome 1 | Qbiogen |
| wcp(2) | chromosome 2 | Qbiogen |
| wcp(3) | chromosome 3 | Qbiogen |
| wcp(4) | Chromosome 4 | Qbiogen |
| wcp(5) | Chromosome 5 | Qbiogen |
| wcp(6) | Chromosome 6 | Qbiogen |
| wcp(8) | Chromosome 8 | Kreatec |
| wcp(9) | Chromosome 9 | Qbiogen |
| wcp(10) | Chromosome 10 | Qbiogen |
| wcp(12) | Chromosome 12 | Qbiogen |
| wcp(13) | Chromosome 13 | Qbiogen |
| wcp(14) | Chromosome 14 | Qbiogen |
| wcp(15) | Chromosome 15 | Qbiogen |
| wcp(16) | Chromosome 16 | Qbiogen |
| wcp(17) | Chromosome 17 | Qbiogen |
| wcp(18) | Chromosome 18 | Kreatec |
| wcp(19) | Chromosome 19 | Qbiogen |
| CEP(1) | 1p11.1q11.1 | Abbott/Vysis |
| LSI EGFR/CEP7 | 7p12(EGFR)/7p11.1q11.1(CEP7) | Abbott/Vysis |
| LSI MYC BAP | 8q24 [MYC 3' (cen) Myc5' (tel)] | Abbott/Vysis |
| LSI BCR/ABL | 9q34(ABL)/22q11(BCR) | Qbiogen |
| LSI IGH/BCL2 | 14q32(IGH)/18q21(BCL2) | Qbiogen |
| LSI DiGeorge/VCFS | 22q11.2 | Qbiogen |
| LSI ATM/P53 | 11q13/17p13 | Abbott/Vysis |
| LSI EWS BAP | 22q12 | Master Diagnostika |
|  |  |  |
| **Locus** | **Genes** | **BAC clones** |
| 1q21.2 | PIAS3 | RP11-767O2 |
|  | *BCA2* | RP11-74F4 |
| 1q25.2 | *LHX4* | RP11-175C8 |
| 2p23.1p22.3 | *BIRC6* | RP11-121M15 |
| 2p16.1 | *REL* | RP11-373L24 |
