# Supplementary material for: High Resolution Genome-Wide Analysis of Chromosomal Alterations in Burkitt's Lymphoma
Source: PLoS One. 2009 Sep 17;4(9):e7089. doi: 10.1371/journal.pone.0007089 (PMC2739276; doi:10.1371/journal.pone.0007089)
Supplement: Table S2 — Clonal Immunoglobulin phenotype linked to chromosomal abnormalities and acquired CNV (0.08 MB DOC) [file pone.0007089.s002.doc]

| Cell lines | CD19 | **surface** | **surface** | **surface** | **intraC*** | **intraC** | **intraC** | **specific** | **IGK** | **IGH** | **IGL** |
| --- | --- | --- | --- | --- | --- | --- | --- | --- | --- | --- | --- |
|  |  | **IgM** | **Kappa** | **Lambda** | **IgM** | **Kappa** | **Lambda** | **Abnormalites** | **Gene** | **Gene** | **Gene** |
| **BL2** | 98% | 100% | neg | 100% | 100% | neg | 100% | t(8 ;22) | -/- | -/- | -/- |
| **BL31** | 87% | 100% | 100% | neg | 100% | 99% | neg | t(8 ;14) | -/- | -/- | +/+ |
| **BL41** | 99% | 100% | 100% | neg | 100% | 100% | neg | t(8 ;14) | -/+ | -/+ | /+ |
| **BL70** | 100% | 100% | 100% | neg | 100% | 100% | neg | t(8 ;14) | -/- | -/+ | +/+ |
| **BL84** | 80% | 96% | neg | 90% | 100% | neg | 99% | t(8 ;22) | -/+ | -/+ | -/- |
| **BL 104** | 100% | 100% | 100% | neg | 100% | 100% | neg | t(8 ;22) | -/+ | -/- | -/- |
| **BL LAL** | 100% | 100% | neg | 100% | 100% | neg | 98% | t(8 ;14) | -/- | -/- | -/- |
| **BLMER** | 100% | 100% | 100% | neg | 100% | 100% | neg | t(8 ;14) | -/+ | -/+ | +/+ |
| **Ly47** | 99% | 82% | neg | neg | 100% | 92% | neg | t(8 ;22) | -/- | -/+ (>IGD) | -/- |
| **Ly91** | 100% | 100% | 61% | neg | 100% | 99% | neg | t(2;8) | -/- | -/- | +/+ |
| **Namalwa** | 97% | 98% | 46% | 92% | 100% | 52% | 94% | t(8 ;14) | -/+ | -/+ | -/+ |
| **OKU** | 80% | 84% | 79% | 18% | 100% | 99% | 61% | t(8 ;22) | -/- | -/+ | -/+ |
| **Ramos** | 97% | 95% | neg | 96% | 100% | neg | 100% | t(8 ;14) | -/+ | -/+ | -/- |
| **Salina** | 100% | 100% | 100% | neg | 100% | 100% | neg | t(8 ;14) | -/+ | -/- | -/+ |
| **Seraphina** | 100% | 94% | neg | 93% | 100% | 100% | 90% | t(8 ;14) | -/+ | -/- | -/- |

*intraC : intracytoplasmic
