# Supplementary figures and images for: High Resolution Genome-Wide Analysis of Chromosomal Alterations in Burkitt's Lymphoma
Source: PLoS One. 2009 Sep 17;4(9):e7089. doi: 10.1371/journal.pone.0007089 (PMC2739276; doi:10.1371/journal.pone.0007089)

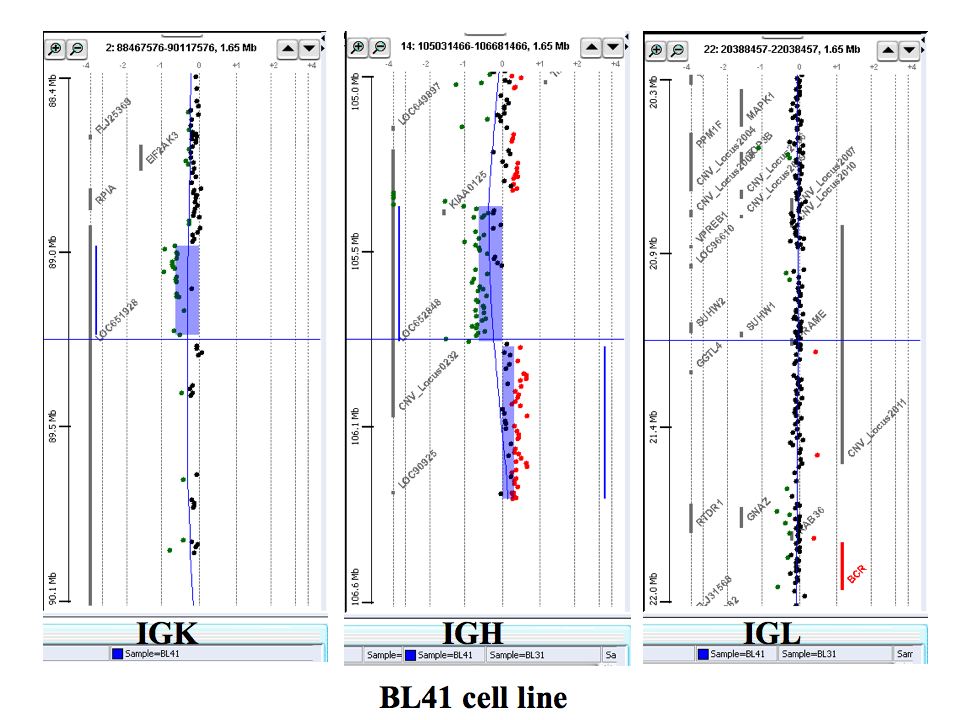

Supplement: Figure S1 — BL41 cell line. (0.11 MB JPG) [file pone.0007089.s003.jpg]

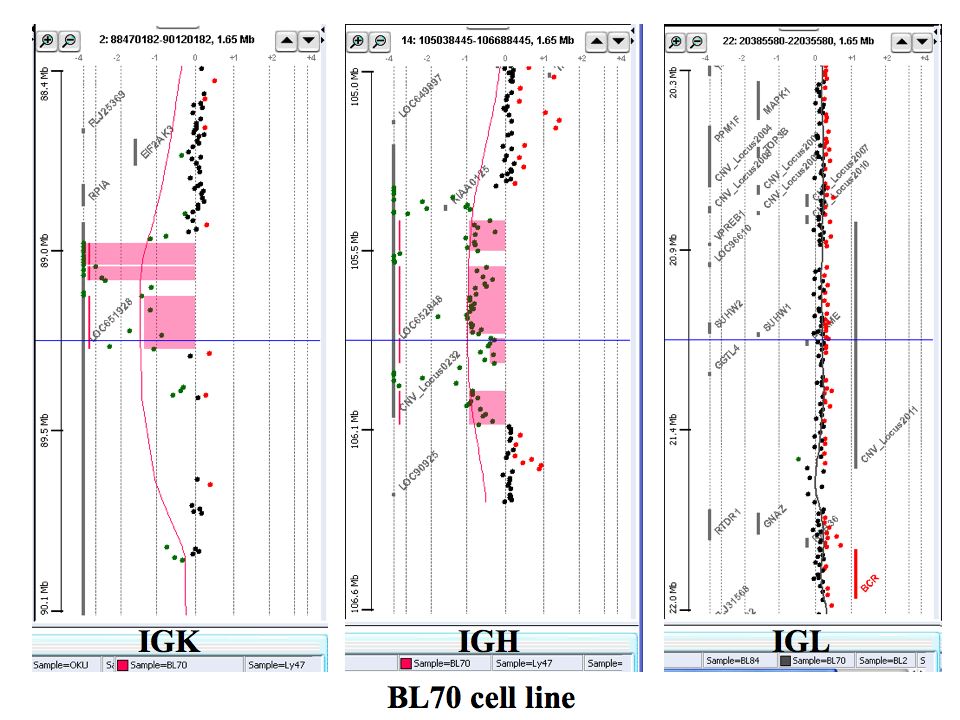

Supplement: Figure S2 — BL70 cell line (0.12 MB JPG) [file pone.0007089.s004.jpg]

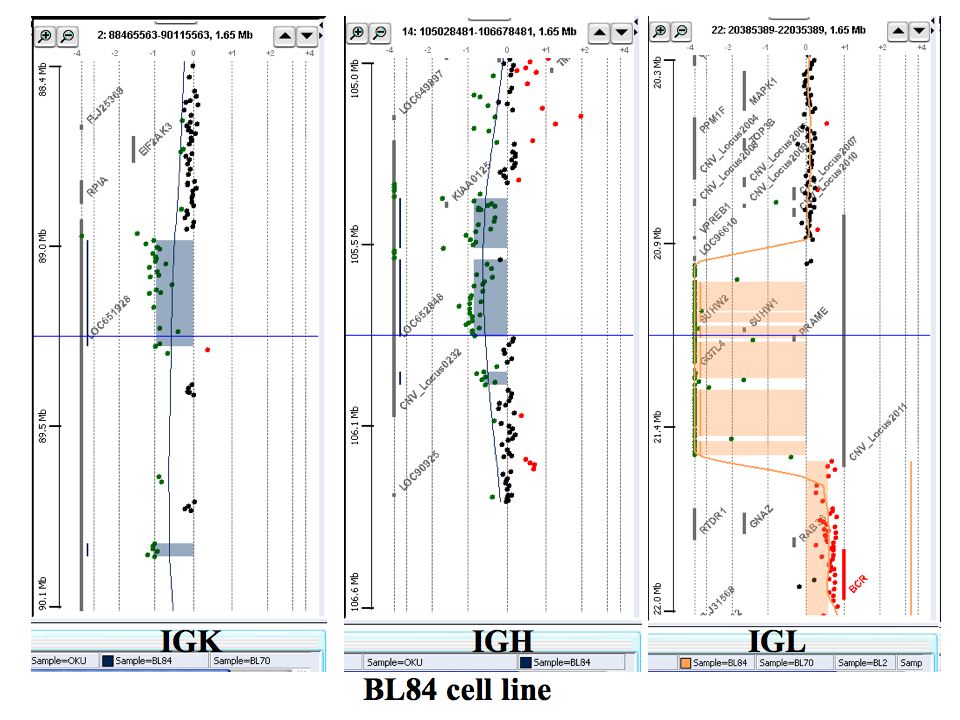

Supplement: Figure S3 — BL84 cell line (0.12 MB JPG) [file pone.0007089.s005.jpg]

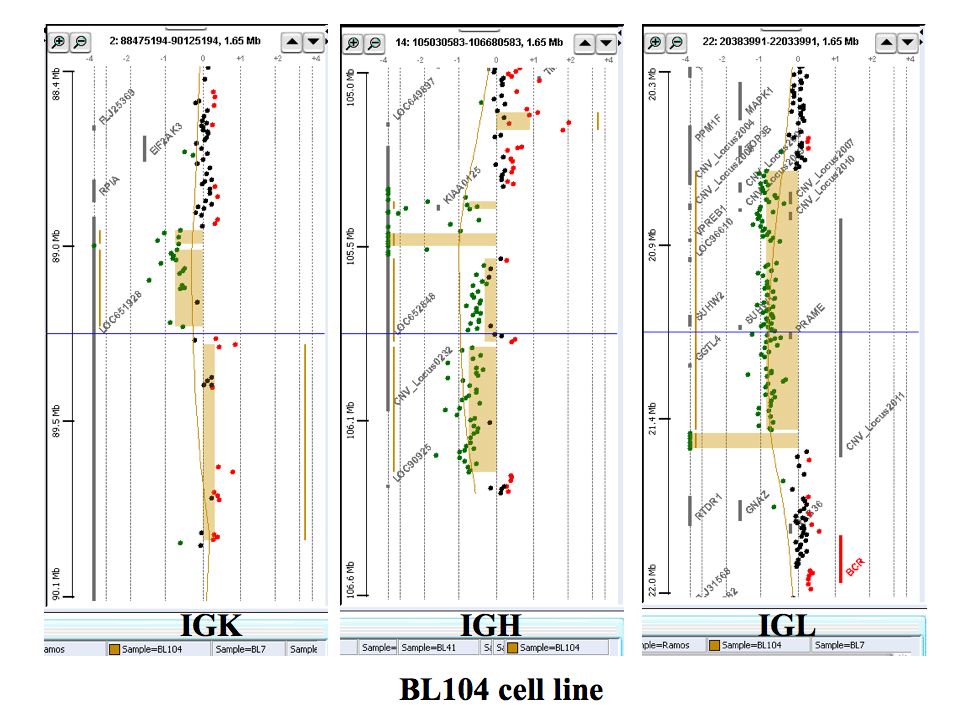

Supplement: Figure S4 — BL104 cell line (0.12 MB JPG) [file pone.0007089.s006.jpg]

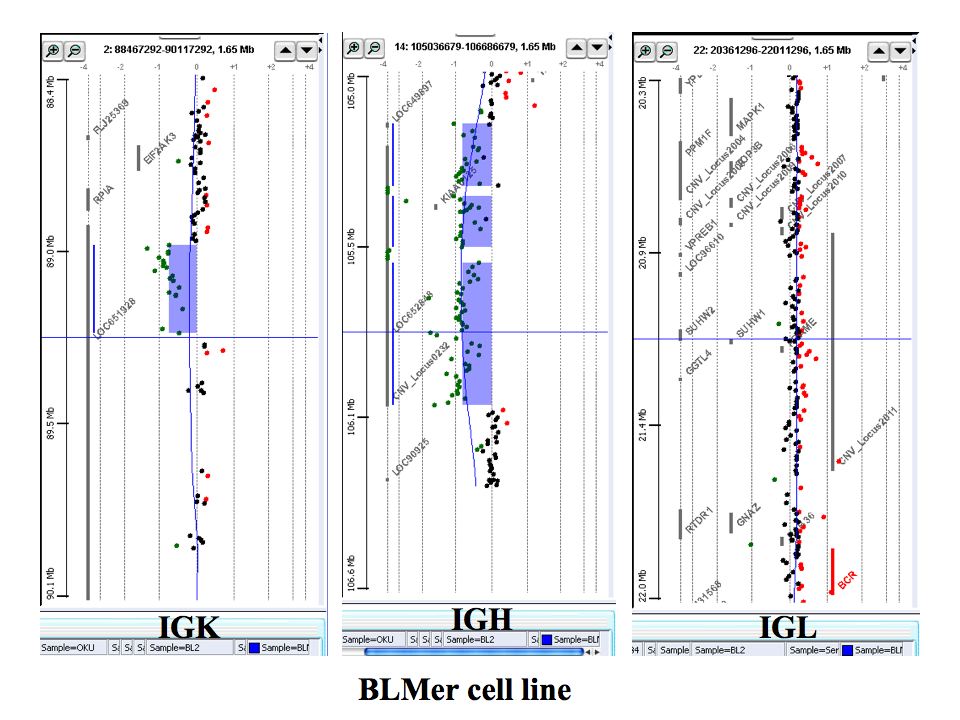

Supplement: Figure S5 — BLMer cell line (0.12 MB JPG) [file pone.0007089.s007.jpg]

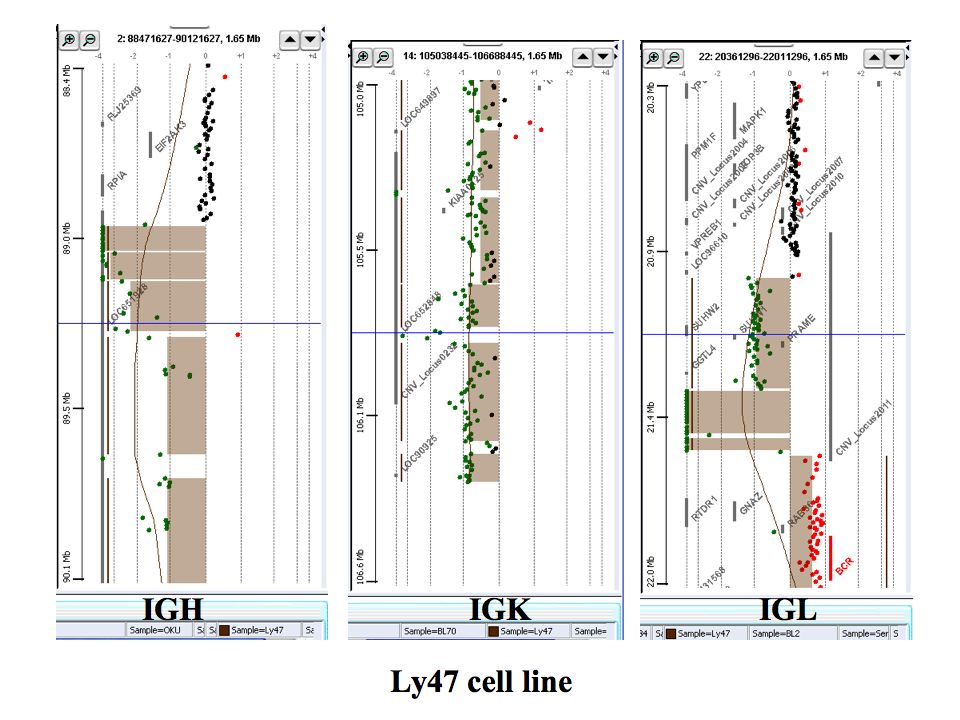

Supplement: Figure S6 — Ly47 cell line (0.11 MB JPG) [file pone.0007089.s008.jpg]

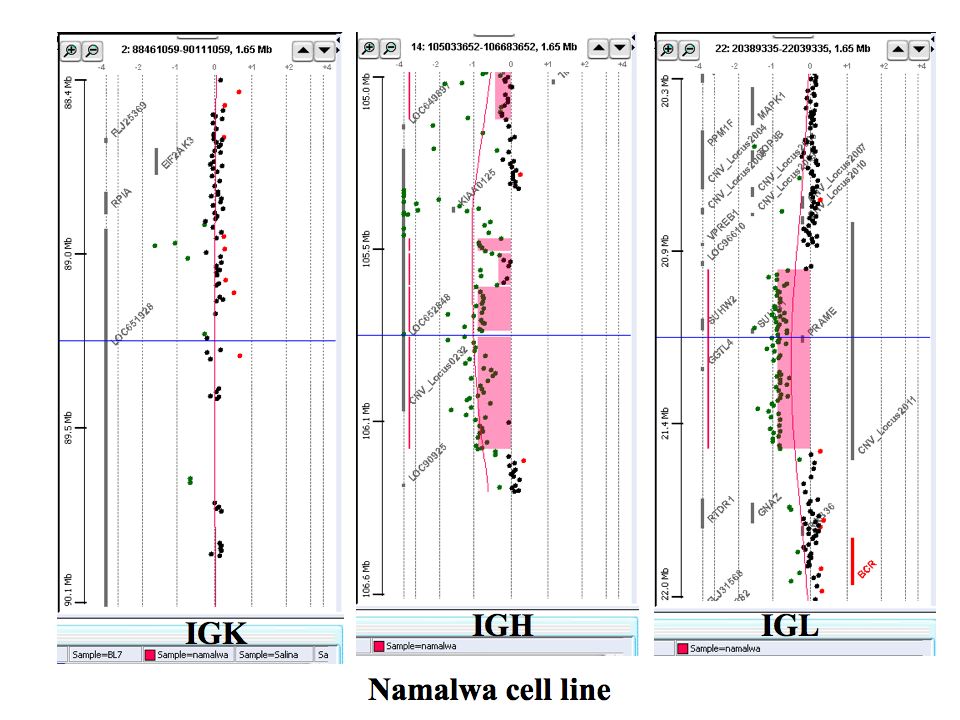

Supplement: Figure S7 — Namalwa cell line (0.11 MB JPG) [file pone.0007089.s009.jpg]

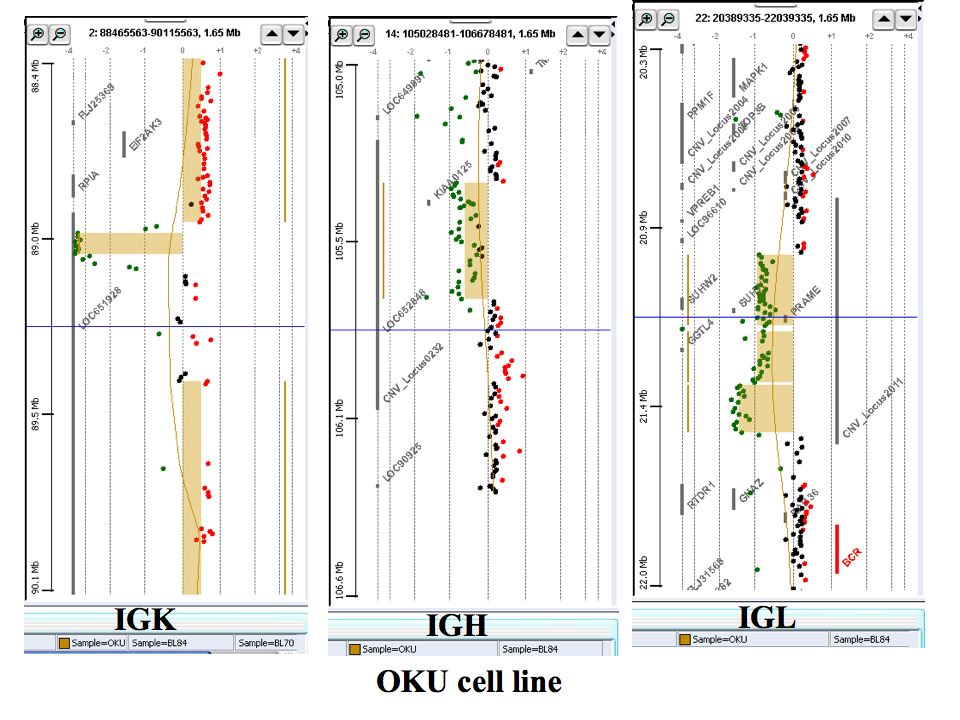

Supplement: Figure S8 — OKU cell line (0.12 MB JPG) [file pone.0007089.s010.jpg]

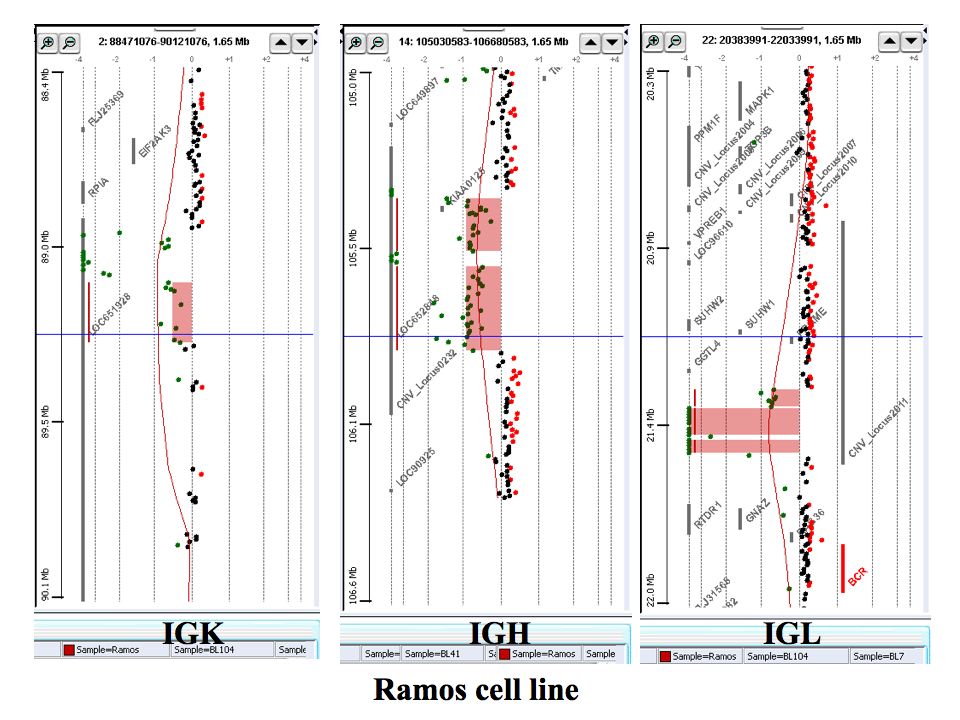

Supplement: Figure S9 — Ramos cell line (0.12 MB JPG) [file pone.0007089.s011.jpg]

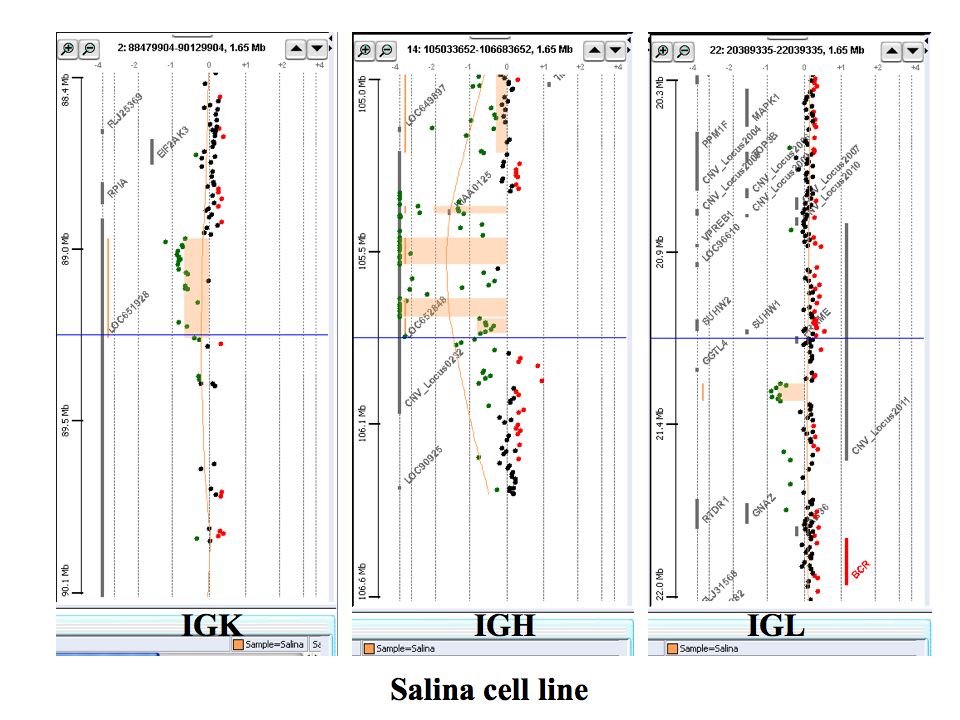

Supplement: Figure S10 — Salina cell line (0.12 MB JPG) [file pone.0007089.s012.jpg]

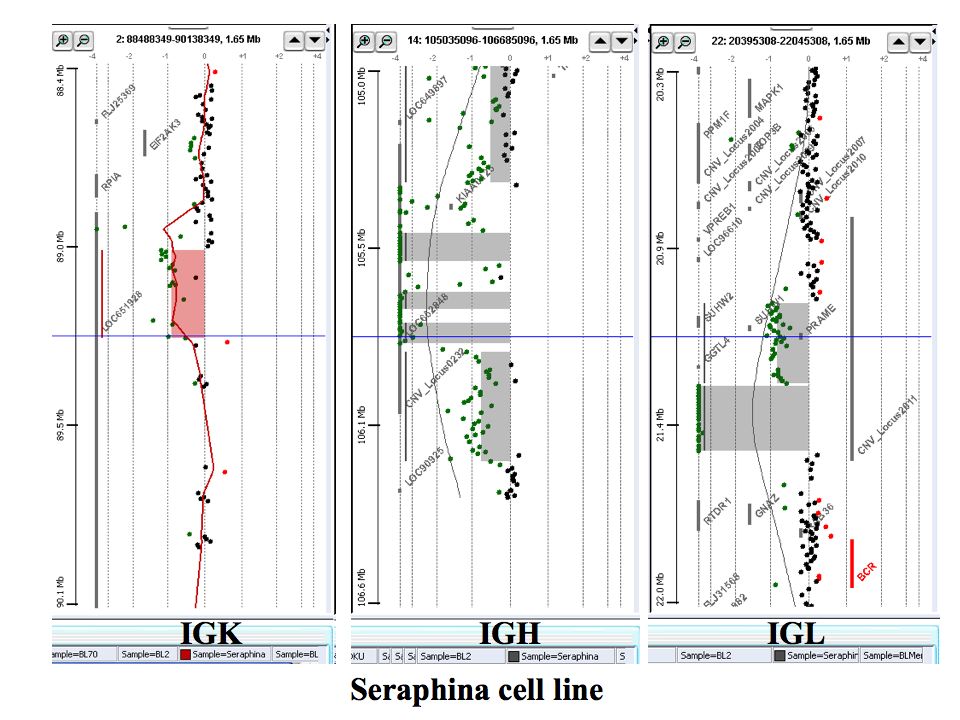

Supplement: Figure S11 — Seraphina cell line (0.12 MB JPG) [file pone.0007089.s013.jpg]
